# Diversity and Complexity of CTXΦ and Pre-CTXΦ Families in *Vibrio cholerae* from Seventh Pandemic

**DOI:** 10.3390/microorganisms12101935

**Published:** 2024-09-24

**Authors:** Xiaorui Li, Yu Han, Wenxuan Zhao, Yue Xiao, Siyu Huang, Zhenpeng Li, Fenxia Fan, Weili Liang, Biao Kan

**Affiliations:** National Key Laboratory of Intelligent Tracking and Forecasting for Infectious Diseases, National Institute for Communicable Disease Control and Prevention, Chinese Center for Disease Control and Prevention, Changbai Road 155, Changping District, Beijing 102206, China; lixiaorui@icdc.cn (X.L.); yuhan9287@gmail.com (Y.H.); zhao-wx@foxmail.com (W.Z.); xiaoyue@icdc.cn (Y.X.); hsy421an@163.com (S.H.); lizhenpeng@icdc.cn (Z.L.); fanfenxia@icdc.cn (F.F.)

**Keywords:** *V. cholerae*, CTXΦ, pre-CTXΦ, RstR, genome structure

## Abstract

CTXΦ is a lysogenic filamentous phage that carries the genes encoding cholera toxin (*ctxAB*), the main virulence factor of *Vibrio cholerae*. The toxigenic conversion of environmental *V. cholerae* strains through CTXΦ lysogenic infection is crucial for the emergence of new pathogenic clones. A special allelic form of CTXΦ, called pre-CTXΦ, is a precursor of CTXΦ and without *ctxAB*. Different members of the pre-CTXΦ and CTXΦ families are distinguished by the sequence of the transcriptional repressor-coding gene *rstR*. Multiple *rstR* alleles can coexist within a single strain, demonstrating the diverse structure and complex genomic integration patterns of CTXΦ/pre-CTXΦ prophage on the chromosome. Exploration of the diversity and co-integration patterns of CTXΦ/pre-CTXΦ prophages in *V. cholerae* can help to understand the evolution of this phage family. In this study, 21 *V. cholerae* strains, which were shown to carry the CTXΦ/pre-CTXΦ prophages as opposed to typical CTX^ET^Φ-RS1 structure, were selected from approximately 1000 strains with diverse genomes. We identified two CTXΦ members and six pre-CTXΦ members with distinct *rstR* alleles, revealing complex chromosomal DNA integration patterns and arrangements of different prophages in these strains. Promoter activity assays showed that the transcriptional repressor RstR protected against CTXΦ superinfection by preventing the replication and integration of CTXΦ/pre-CTXΦ phages containing the same *rstR* allele, supporting the co-integration of the diverse CTXΦ/pre-CTXΦ members observed. The numbers and types of prophages and their co-integration arrangements in serogroup O139 strains were more complex than those in serogroup O1 strains. Also, these CTXΦ/pre-CTXΦ members were shown to present the bloom period of the CTXΦ/pre-CTXΦ family during wave 2 of the seventh cholera pandemic. Together, these analyses deepen our comprehension of the genetic variation of CTXΦ and pre-CTXΦ and provide insights into the evolution of the CTXΦ/pre-CTXΦ family in the seventh cholera pandemic.

## 1. Introduction

*Vibrio cholerae* is a Gram-negative bacterium prevalent in aquatic habitats and the causative agent of cholera, posing a significant threat to public health and economic prosperity [1]. In 2022, 472,697 cases and 2349 deaths in 44 countries were reported to the World Health Organization [2,3]. Although *V. cholerae* encompasses more than 200 serogroups, only serogroups O1 and O139 can actively colonize the human gastrointestinal tract and cause diseases [4,5]. Serogroup O1 can be further classified into two biotypes, class and El Tor (ET) [6].

The main virulence factors of *V. cholerae* are cholera toxin (CT) and toxin-coregulated pilus (TCP, a homopolymer of the major pilin subunit TcpA) [7]. The CT genes *ctxA* and *ctxB* are carried in the lysogenic filamentous phage CTXΦ, which integrates into the bacterial chromosome [8,9]. The infection and lysogenization of CTXΦ make *V. cholerae* pathogenic [6,10,11,12]. The 7.0 kb CTXΦ prophage genome is composed of two distinct modules: the 2.4 kb repeat sequence (RS) 2 (which harbors *rstR*, *rstA*, and *rstB* genes) and the 4.6 kb core structure region (which includes *psh*, *cep*, *pIII^CTX^*, *ace*, *zot*, and *ctxAB* genes) [13]. CTXΦ without *ctxAB* in the core structure region is called pre-CTXΦ, which is around 5.8 kb [13]. While class CTXΦ types are integrated individually or as truncated copies, ET CTXΦ types are usually arranged in tandem or combined with related genetic factors [11,12]. In ET strains, CTXΦ is typically arranged in tandem with the RS1 satellite phage genome in the large chromosome (chromosome 1) [12]. The approximately 2.7 kb RS1 genome contains *rstR*, *rstA*, and *rstB* genes, which are present in RS2 and allow phage replication and integration, and an additional ORF termed *rstC*, which maintains CTXΦ lysogeny [12,13]. RstA nicks the intergenic sequence 1 (Ig-1) upstream of *rstR*, as well as the Ig-1 of the adjacent CTXΦ prophage or RS1 downstream of the CTXΦ prophage genome, thereby releasing a closed circular ssDNA and achieving replication by a rolling-circle mechanism [12]. Therefore, the CTXΦ in the class biotype does not produce CTXΦ DNA outside the chromosome and does not produce viral bodies, while the CTXΦ in the ET biotype can be infectious [12]. The presence of RS1 or the continual presence of two or more CTXΦ prophages (i.e., the structural diversity of CTXΦ genomes) is a crucial factor in determining whether the CTXΦ can replicate and in evaluating its pathogenicity.

RstR is an allele-specific repressor that regulates CTXΦ replication by inhibiting the activity of the corresponding promoter [14,15,16]. RstR binds to the ig-2 region of the *rstAB* promoter and inhibits *rstAB* expression in CTXΦ of the same type but not the other types. Eleven *rstR* types have been identified to date based on their allelic sequences: *rstR^class^* [16], *rstR^ET^* [10], *rstR^calc^* [17], *rstR^232^* [18], rstR-4*, rstR-4** [19], *rstR^5^* [19], *rstR^6^* [20,21], *rstR^ZHJ^* [22], *rstR^JS^* [19], and *rstR^ZJ^* [23]. The divergence of *rstR* genes provides the molecular basis for the specificity of CTXΦ immunity [24]. In addition, *rstR* and *ctxB* have been used to distinguish more detailed variants of CTXΦ [25,26,27]. Therefore, RstR is crucial for discovering new CTXΦ variants and inhibiting the expression and lysogenic conversion of CTXΦ.

Special forms of CTXΦ, such as nct-CTXΦ [28] or pre-CTXΦ, do not carry *ctxAB* but contain other genes considered to be CTX prophage sequences [29], such as *ace* and *zot*, which retain the potential to induce cholera-like symptoms and have potential significance for cholera pathogenesis. Due to the lack of *ctxAB*, *rstR* alleles play a decisive role in identifying pre-CTXΦ. In this study, we aimed to reveal the contribution of the CTXΦ/pre-CTXΦ families in the evolution and disease prevalence of *V. cholerae* by describing their diversity, co-integration patterns, and the role of RstR in CTXΦ expression and lysogenic transformation. Based on the diversity of *rstR* alleles, we selected 21 *V. cholerae* strains from approximately 1000 specimens from different sources and assessed the temporal and spatial distribution, copy number variation, and co-integration patterns of pre-CTXΦ and CTXΦ, as well as the role of RstR in CTXΦ expression and lysogenic transformation. The gene organization structure analysis showed that the strains in wave 2 of the seventh epidemic have a complex CTXΦ/pre-CTXΦ family form. The co-integration arrangements of CTXΦ/pre-CTXΦ prophages in serogroup O139 strains were found to be more complex than that in serogroup O1 strains. The *rstAB* promoter activity analysis confirmed that the RstR allele specifically inhibits the expression of *rstAB* in different CTXΦ. We assumed that during wave 2 of the seventh cholera pandemic, which was caused by serogroup O139 strains in the Chinese mainland, pre-CTXΦ promotes the diversity of the CTXΦ family by serving as an intermediate pool of *rstR* alleles. Together, these results contribute a deeper comprehension of the genetic diversity of the CTXΦ family, the evolution between CTXΦ and pre-CTXΦ, and the phage immunity mediated by RstR.

## 2. Materials and Methods

### 2.1. Bacterial Strains and Culture Conditions

Thousands of *V. cholerae* strains have been maintained in our laboratory since 1961. In this study, 21 strains were selected for whole-genome sequencing (WGS) based on the abundance of *rstR* types and the presence of *ctxAB* determined by a previous draft genome sequencing analysis. Colonies were identified by agglutination tests with antisera from serogroups O1 and O139 and oxidase tests. Serotyping was also performed using different types of serum kits (Serotest, Thailand) [30]. The positive strains (serogroups O1 and O139 strains) were validated using real-time polymerase chain reaction (PCR), with primers targeting the O1-specific *wb* and O139-specific *wbf* genes [31].

The *V. cholerae* and *Escherichia coli* strains utilized in this study are listed in Table S1. All strains were grown at 37 °C in Luria Bertani (LB) medium (Oxoid, Basingstoke, UK). Antibiotics were added as needed at the following final concentrations: ampicillin (Amp), 100 μg/mL; chloramphenicol (Cm), 10 μg/mL for *E. coli* and 2.5 μg/mL for *V. cholerae*.

### 2.2. Recombinant Plasmid Construction

PCR was performed to amplify eight *rstR* alleles (ET, Class, 232, 5, 6, ZJ, ZHJ, and Calc) with specific primers and *V. cholerae* genomic DNA templates. PCR fragments were then individually cloned into linearized pBAD24 plasmid using a pEASY-Uni Seamless Cloning and Assembly Kit (TransGen Biotech, Beijing, China), resulting in the formation of eight recombinant plasmids: pB-*rstR*^ET^, pB-*rstR*^class^, pB-*rstR*^232^, pB-*rstR*^5^, pB-*rstR*^6^, pB-*rstR*^ZJ^, pB-*rstR*^ZHJ^, pB-*rstR*^Calc^. Based on the same procedure above, PCR fragments of different ig-2 promoters were amplified and cloned into pBBR-*lux* reporter plasmid to generate eight recombinant plasmids: pBR-ig2-ET, pBR-ig2-class, pBR-ig2-232, pBR-ig2-5, pBR-ig2-6, pBR-ig2-ZJ, pBR-ig2-ZHJ, and pBR-ig2-Calc. The plasmids and primers used in this study are listed in Table 1 and Table 2.

### 2.3. Luminescence Assay

To evaluate the regulative effect of different RstR alleles to the corresponding *rstAB* expression, recombinant pBAD24 plasmids containing *rstR* genes and recombinant pBBR-*lux* plasmids containing ig-2 sequences were introduced into *E. coli* JM109, and the luminescence activities were measured to assess the ability of the *rstR* gene to inhibit the corresponding ig-2 promoter. An empty pBAD24 plasmid was coexpressed with each pBR-ig2 plasmid in JM109, which served as negative controls (Table 2 and Table S1).

The cultures of strains harboring different combinations of pB-*rstR* and pBBR-*lux* reporter plasmids were diluted (1:100) in fresh LB medium and grown at 37 °C with shaking. Then, 200 μL culture was transferred hourly to a 96-well tissue culture plate (#3917, Costar, Corning, NY, USA), and the values of luminescence and OD_600_ were measured using a plate reader (Infinite M200 Pro, Tecan, Grödig, Austria). Relative light units (Luminescence/OD_600_ values) were calculated as described previously [32]. Data are presented as means ± standard deviations of three biological replicates performed in triplicates.

### 2.4. WGS

Chromosomal DNA was extracted using a Wizard Genomic DNA Purification Kit (Promega, Madison, WI, USA) and sequenced on a high-throughput sequencing platform (Pacific Biosciences, Menlo Park, CA, USA). Raw sequencing data were subject to quality control by Fastp (v0.19.6, HaploxBiotechnology Co, Ltd, Shenzhen, China) tool to remove low-quality reads and adapters, correct sequencing errors, and deduplicate redundant data [33]. Clean reads were then assembled de novo using SPAdes (v3.15.5, St. Petersburg State University, Russia, St. Petersburg Academic University, Russia, University of California, San Diego, CA, USA) software. To increase the accuracy of the assembled genome, four rounds of iterative error correction were performed using an in-house script with the clean Illumina data.

### 2.5. Genetic Element Analysis

Functional genes (*ctxAB* and *rstA/B/C/R*) and genes of pathogenicity islands (VPI, VSP-1, and VSP-2) were identified using Prokka (v1.12, University of Melbourne, Melbourne, Australian) software. For comparative analysis of pathogenicity islands, BLAST (https://www.ncbi.nlm.nih.gov/blast, accessed on 13 May 2023) was used to map query sequences to the reference sequences of strain N16961 (Table S3), allowing the retrieval and comparison of whole genomes [25]. The accession numbers of other strains are listed in Table S1. SNP-based phylogenetic analysis of *V. cholerae* genomes was conducted using kSNP version 3.0 with default settings. Representative genomes were selected from clusters, and a phylogenetic tree was built using R package [26]. In silico multi-locus sequence typing (MLST) was performed using the screen-Blast-MLST function of the Pathogenomics R package (https://github.com/UCL-CTMA/Pathogenomics, accessed on 11 November 2023), which requires 100% sequence identity and coverage for allele assignment.

### 2.6. Statistical Analysis

Statistical analysis and data visualization were carried out using GraphPad Prism 9. Statistical significance was determined using an unpaired two-tailed *t*-test.

## 3. Results

### 3.1. Complexity of CTXΦ/Pre-CTXΦ Alleles and Their Arrangements in the Chromosomes of V. cholerae Strains

**Figure 1:**
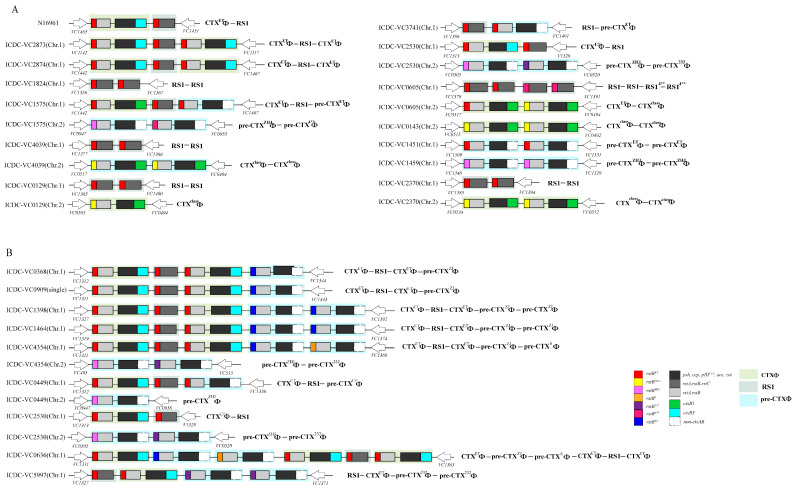
Genetic structure of CTXΦ (6.9 kb) and pre-CTXΦ (5.8 kb) in serogroups O1 (**A**) and O139 (**B**) strains. The classification is primarily based on functional domains. RstR downregulates the *rstA* promoter. RstA and RstB play crucial roles in phage replication and integration. The structural proteins Psh, Cep, pIII, Ace, and Zot are essential for the morphology and assembly of the phage. The dark black solid squares represent the structural proteins of CTXΦ. The colored squares represent different *rstR* alleles (ET, class, Calc, 4**, 6, 232, ZJ, and ZHJ), while the hollow squares indicate the deletion of *ctxAB*. The fluorescent green and lake blue solid squares represent *ctxB1* and *ctxB3*, respectively. The direction of the hollow arrow indicates the direction of gene transcription, and the corresponding numbers indicate the specific locations of the genes on the chromosome. Except for the special marking RS1^4**^, all other RS1 (2.7 kb) types are RS1^ET^.

The seventh pandemic ET strain N16961 was the firstly sequenced *V. cholerae* strain, which has the typical CTXΦ-RS1 prophage array in its chromosome 1 (Chr. 1) [34]. From the genomes of the seventh pandemic and serogroup O139 strains we sequenced, CTXΦ/pre-CTXΦ-RS1 arrays different from the array in N16961 were identified. The characteristics of these CTXΦ/pre-CTXΦ-RS1 arrays include more *rstR* sequence types other than El Tor type, unequal copies of *zot* and *rstC* (suggesting unequal copies between CTXΦ/pre-CTXΦ and RS1), possible possession of pre-CTXΦ, and the presence of only RS1/RS2 elements in nontoxigenic strains. We screened a total of 31 complete CTXΦ sequences, including two CTXΦ members (CTX^class^Φ, 12.50%; CTX^ET^Φ, 42.86%) and six pre-CTXΦ members carrying different *rstR* alleles (pre- CTX^ET^Φ, 8.93%; pre-CTX^ZHJ^Φ, 8.93%; pre-CTX^232^Φ, 7.14%; pre-CTX^ZJ^Φ, 14.29%; pre-CTX^6^Φ, 3.57 %; pre-CTX^4**^Φ, 1.78%). Among them, CTX^ET^Φ has the highest detection rate, and the lowest is pre-CTX^4**^Φ. Also, complex chromosomal DNA integration patterns and arrangements of different CTXΦ/pre-CTXΦ prophages were observed in these strains, indicating that these strains may have unusual prophage genome structures of CTXΦ/pre-CTXΦ (Figure 1) but not CTXΦ-RS1 array. Complete genome sequences of these strains were obtained by the combined assembly with short-read sequencing and long-read sequencing of NGS to clearly reveal the gene organization and genomic arrays of CTXΦ/pre-CTXΦ and RS1 in each strain (Figure 1). We further designed primers specific to different *rstR* sequence types (Table S2), including *rstR^class^*, *rstR^ET^*, *rstR^calc^*, *rstR^5^*, *rstR^6^*, *rstR^232^*, rstR^4**^, *rstR^ZJ^*, and *rstR^ZH^*^J^, and performed PCR to detect the *rstR* sequence types in these strains.

For the 21 strains possessing unusual CTXΦ/pre-CTXΦ arrays, 13 strains belong to serogroup O1 and the remaining belong to serogroup O139. The arrays of CTXΦ/pre-CTXΦ and RS1 are presented in Figure 1. In the O1 strains, five strains carry the CTX^class^Φ genome, and all these prophage genomes are integrated in chromosome 2 (Chr. 2) (Figure 1A and Table 1). Strain ICDC-VC0129 has one copy of the CTX^class^Φ genome but the other three O1 strains (ICDC-VC0143/2370/4039) have two tandem copies. Almost all of them carry two tandem RS1 elements in Chr. 1. An exceptional case was strain ICDC-VC0605, which has a CTX^ET^Φ-CTX^class^Φ array in Chr. 2. and shows a hybrid array of members of the CTXΦ family. Four O1 strains have CTX^ET^Φ/pre-CTX^ET^Φ prophage genomes in Chr. 1 but have different member arrays of CTXΦ (Figure 1A and Table 1): two strains (ICDC-VC2873 and ICDC-VC2874) have an extra CTX^ET^Φ copy followed with the typical CTX^ET^Φ-RS1 array; strain ICDC-VC2530 has the typical CTX^ET^Φ-RS1^ET^ array in Chr. 1 but has two additional, different prophage genomes of pre-CTX^ZHJ^Φ and pre-CTX^232^Φ, which are integrated in Chr. 2 in tandem; strain ICDC-VC1575 has an extra copy of pre-CTX^ET^Φ except for CTX^ET^Φ-RS1 in Chr. 1 and has two different copies of prophage genomes of pre-CTX^ZJ^Φ and CTX^4**^Φ in Chr. 2. Strain ICDC-VC1575 also had a hybrid CTXΦ in Chr. 1, which carries the *ctxB1* of the class biotype (Table S4). Strain ICDC-VC3741 is a nontoxigenic strain and has RS1-pre-CTX^ET^Φ genome; the other two strains (ICDC-VC1451 and 1459) are also *ctxAB*-negative and carry two tandem copies of pre-CTXΦ, but they belong to different members of pre-CTXΦ (pre-CTX^ET^Φ and pre-CTX^ZHJ^Φ, respectively). Strain ICDC-VC1824 has an exceptive array which has only RS1^ET^ in Chr. 1 (Figure 1A).

Eight serogroup O139 strains present more complex CTXΦ/pre-CTXΦ arrays and members in the CTXΦ family (Figure 1B and Table 1). Five strains have the basic array component of CTX^ET^Φ-RS1^ET^-CTX^ET^Φ. Among them, strains ICDC-VC0368 and 1398 have one or two additional copies of pre-CTX^ZJ^Φ in Chr. 1; strains ICDC-VC1464 and 4354 have additional pre-CTX^ZJ^Φ and pre-CTX^6^Φ; strain ICDC-VC1464 have additional pre-CTX^ZJ^Φ and pre-CTX^6^Φ; strain ICDC-VC4354 has pre-CTX^ZJ^Φ, pre-CTX^6^Φ, pre-CTX^ZHJ^Φ and pre-CTX^ZJ^Φ integrated in Chr. 2; they represent the most complex hybrid integration of CTXΦ members; strain ICDC-VC0636 has CTX^ET^Φ and two copies of pre-CTX^ZJ^Φ genomes, which are integrated into the upstream of CTX^ET^Φ-RS1-CTX^ET^Φ (Figure 1B and Table 1). Strain ICDC-VC0449 has an exceptive array of the CTX^ET^Φ-RS1^ET^-pre-CTX^ET^Φ, in which the second CTXΦ prophage is pre-CTX^ET^Φ. Strain ICDC-VC5997 has a RS1^ET^-CTX^ET^Φ, which is followed with two copies of pre-CTX^232^Φ, with the RS1 element located in the upstream of CTXΦ prophages. Strain ICDC-VC0909 exhibits a single chromosome in which two chromosomes have been merged. It has a prophage array of CTX^ET^Φ-RS1^ET^-CTX^ET^Φ-pre-CTX^ZJ^Φ, which is located in the corresponding site in Chr. 1.

### 3.2. Phylogeny of V. cholerae Strains Carrying CTXΦ/Pre-CTXΦ Prophages with Unusual Arrangements

**Figure 2:**
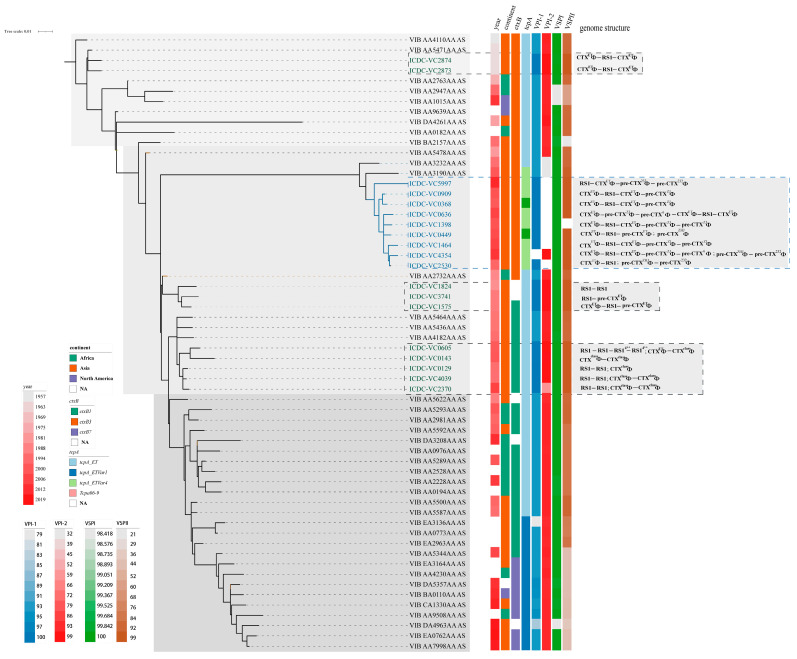
Phylogenetic analysis of 21 *V*. *cholerae* strains from three pandemic waves and other publicly available O1 clones (Table S5). Waves 1, 2, and 3 are represented by different shades of gray (light, medium, and dark represent waves 1, 2, and 3, respectively). Serogroup O139 strains are shown in blue. In addition to the 21 strains used in this study, the other 42 strains originated from different countries. The left side of the tree shows metadata, gene information, and CTXΦ/pre-CTXΦ structure. Each column on the right side represents a specific gene, and the light squares denote the presence of gene alleles. The three dashed boxes of different colors represent different waves (waves 1, 2 and 3). The tree was constructed from SNPs from the reference genome of N16961 strain, and the pre-7PET strain VIB_AA4110AA_A was used as an outgroup.

We constructed a phylogenetic tree based on the seventh pandemic strains and serogroup O139 strains to present the phylogenic position of the 21 strains used in this study. The seventh pandemic strains were selected from EnteroBase (https://enterobase.warwick.ac.uk/species/index/vibrio, accessed on 20 April 2023). A total of 42 strains were included in the phylogenetic analysis, combined with the 21 strains used in this study (Figure 2). These strains also formed a three-wave phylogeny structure [35]. Eight serogroup O139 strains from this study and two O139 strains obtained from EnteroBase formed an independent branch in wave 2. The 21 O1 and O139 strains with unusual integrations of CTXΦ/pre-CTXΦ are shown to concentrate in the wave 2 sublineage and exhibit a high degree of complexity. Serogroup O139 strains have more complex CTXΦ/pre-CTXΦ arrangements in chromosomes compared to O1 strains.

The analysis of the O1 strains revealed the presence of three distinct genomic clones (Figure 2). The ICDC-VC2873 and 2874 strains exhibit identical CTX^ET^Φ-RS1^ET^ arrays and are situated within wave 1. These strains also exhibit the expected arrays of the El Tor biotype of CTXΦ. Strains ICDC-VC1575, 3741, and 1824 are observed to cluster together and show a genome deletion of CTX^ET^Φ and pre-CTX^ET^Φ. Five strains carrying (ICDC-VC0605/2370/0128/0143/4039) CTX^class^Φ prophages formed another clone, showing their close phylogenetic relationship. Most strains have two copies of CTX^class^Φ, possibly resulting from the prophage amplification, except one has one copy and another has a substitution of the CTX^ET^Φ genome (ICDC-VC0605).

Among the phylogenetic tree, the sequence type of toxin gene *ctxB* was found to experience a shift during wave 2. In the strains belonging to wave 1 and the early stage of wave 2, which include the O139 strains, the *B3* type of *ctxB* was identified. In contrast, in the strains belonging to wave 2 and carrying CTX^class^Φ, the *B1* type of *ctxB* was identified. However, in strain ICDC-VC1575 carrying the El Tor type of *rstR*, the *B1* type of *ctxB* was also found, indicating a hybrid CTX phage which might be the transitional allele of CTXΦ. In the strains belonging to wave 3, the *B1* and *B7* types of *ctxB* were identified [33], and the latter type was shown to have appeared in recent years (Figure 2 and Table S4). TcpA is the main subunit of TCP, which serves as the receptor of CTXΦ. The types of *tcpA* gene in serogroup O139 strains were found to be more variable than O1 strains. For example, the dominant type *tcpA*_ET in serogroup O139 strains was temporarily replaced with the *tcpA*_ETVAR4 in wave 2 strains, followed by returning to the dominant type, and finally being replaced with the *tcpA*_ETVAR1 in wave 3 strains (Figure 2 and Table S2).

Genomic analysis demonstrated that the O139 strains were highly divergent and distinct from the O1 strains. Further, the genetic diversity of toxigenic and nontoxigenic O139 strains was higher than that of O1 strains. Also, CTXΦ in wave 2 strains was highly complex. These observations suggest that O139 strains caused an outbreak in the chinses mainland during wave 2, when CTXΦ and pre-CTXΦ were functionally active, with frequent genetic exchange between strains, resulting in the coexistence of multiple family members. The members and co-integration arrangements of CTXΦ/pre-CTXΦ prophages in serogroup O139 strains were found to be more complex than those in serogroup O1 strains. Additionally, the CTXΦ/pre-CTXΦ prophage family exhibited a bloom period during wave 2 of the cholera pandemic.

### 3.3. Evaluation of the Inhibition Effects of Different rstR Alleles on the Homotypic and Heterotypic ig-2-Containing Promoters

**Figure 3:**
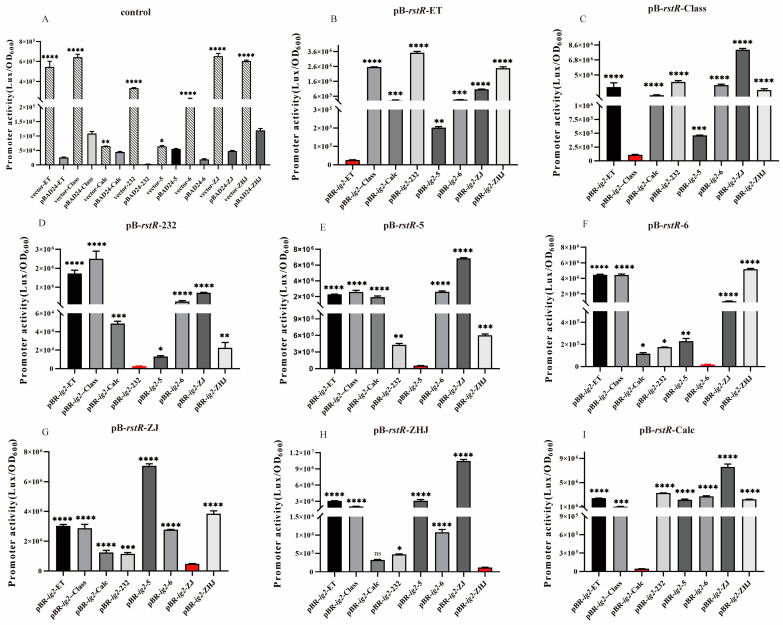
Different RstR alleles specifically repress their corresponding *rstA* (ig-2) promoters. Under identical culture conditions, the activity of each ig-2 promoter is regulated by the expression of the corresponding RstR. (**A**) Eight pBBR-*lux* reporter plasmids were constructed for blank control group experiments to demonstrate the feasibility of plasmid construction. (**B**–**I**) Analysis of promoter activity using a pBBR-*lux* reporter plasmid fused with different *rstA* promoters and a pBAD24 plasmid containing an *rstR* allele (ET, class, 232, 5, 6, ZJ, ZHJ and Calc, respectively). Each *rstR* allele specifically inhibited the luminescence of the corresponding promoter, indicating the inhibition of *rstAB* expression. Data are means and standard deviations of three independent experiments * *p* < 0.0143, ** *p* < 0.0028, *** *p* < 0.002, **** *p* < 0.0001 ( * if the *p*-value was less than 0.05, ** if the *p*-value was less than 0.01, *** if the *p*-value was less than 0.001, **** if the *p*-value was less than 0.0001, and ns for no significant difference).

RstR mediates immunity to CTXΦ superinfection through inhibiting transcription of *rstAB* by binding to the ig-2 region of the same CTXΦ genomes [16]. In this study, we identified seven *rstR* alleles (ET/class/4**/232/ZJ/ZHJ/6) in CTXΦ and pre-CTXΦ among the 21 strains (Table 1). Using recombinant reporter vectors carrying different ig-2 promoters and the pBAD24 plasmid containing the corresponding *rstR* allele or the empty pBAD24 plasmid, the inhibition effects of the different *rstR* alleles were verified (Figure 3A and Table S1). The results showed that the effects of the isotypic inhibition by *rstR* alleles differed among different groups. The promoter activities of *rstR*/ig-2 groups containing ZJ/ZHJ types of *rstR* alleles were higher than those of *rstR*/ig-2 groups containing other types of *rstR* alleles, indicating that pre-CTX^ZJ^Φ and pre-CTX^ZHJ^Φ have higher expression levels of *rstAB*, more phage assembly and formation, and more opportunities for interspecific horizontal gene transfer. In contrast, the promoter activities of *rstR*/ig-2 groups containing Calc/5 types of *rstR* alleles were lower than those of *rstR*/ig-2 groups containing other types of *rstR* alleles (Figure 3A).

Then, each pB-*rstR* plasmid was coconjugated with different pBR-ig2 plasmids (Table 2). The promoter activity of the conjugation combination of pB-*rstR*-ET with pBR-ig-2-ET was much lower compared to those of conjugation combinations of pB-*rstR*-ET and pBR-ig-2 containing other ig-2 promoters (Figure 3B and Table 2), indicating the specific inhibition of *rstR^ET^* to its own ig-2-containing promoter. The similar homotypic inhibition of *rstR* to its ig-2-containing promoter was also observed in the coconjugation groups of the other types of *rstR*, in which the effect of heterotypic inhibition of each *rstR* was lower than that of homotypic inhibition (Figure 3C–I and Table 2). The inhibitory activity of homotypic inhibition was highest in pre-CTX^ZJ^Φ (*rstR^ZJ^*, exhibiting a 13.4-fold increase compared to the other five *rstR* types). Furthermore, our analysis clearly showed that the detection rate of pre-CTX^ZJ^Φ (*rstR^ZJ^*) in the genomes of the serogroup O139 strains was as high as 75% (Table 2). Therefore, we speculated that pre-CTX^ZJ^Φ may achieve high activity of co-infection and integration with other strains because of its abundance and the strong RstR-mediated self-inhibition or immunity, leading to its widespread presence in the specific genome arrangements of the CTXΦ family. This adaptation may be associated with the diminished transformation rate of the CTXΦ phage containing strains and the virulence of *V. cholerae*, but the specific mechanism still needs to be explored.

No cross-immunity exists between different *rstR* alleles, allowing co-infection with CTXΦ and pre-CTXΦ phages in *V. cholera* strains. The fact that RstR mediates CTXΦ specificity prevents CTXΦ infection among strains having the same *rstR* type (that is, promotes CTXΦ immunity), as strains carrying pre-CTXΦ cannot be infected with a CTXΦ phage containing the same *rstR* type. This selection mechanism prevents the toxigenic conversion of CTXΦ family members’ strains harboring the same *rstR* alleles.

## 4. Discussion

Bacteriophages can convert their bacterial hosts from nonpathogenic strains to pathogenic strains through phage conversion by providing the host with phage-encoded virulence genes [36]. Toxigenic *V. cholerae* isolates carry the *ctxAB* genes encoded by a lysogenic filamentous phage CTXΦ [12]. The different types of CTXΦ were determined based on *rstR* sequence differences, and these members constitute the CTXΦ family [22,23,32]. Nevertheless, the form of pre-CTXΦ that lacks *ctxAB* also existed [28]. Furthermore, multiple members of CTXΦ can coexist in the chromosome of the same strain [29], resulting in a complex CTXΦ family within the *V. cholerae* species. Because of different copy numbers of different genetic elements, it is challenging to construct comprehensive physical maps of *V. cholerae* genomes using second-generation WGS. Therefore, in this study, we constructed a complete map of the *V. cholerae* genomes from 21 strains using WGS [22,37]. In this study, the observation was made that a more complex structure of the CTXΦ/pre-CTXΦ family together with chromosomal genes was present in the seventh pandemic strains. The completed map of *V. cholerae* genomes illustrates the diversity of the CTXΦ/pre-CTXΦ family members, the co-integration of multiple members in the chromosomes, and the complex genomic arrangements. These phenomena mainly occurred during wave 2, indicating that the CTXΦ/pre-CTXΦ family was undergoing active development and transfer. Furthermore, the co-integration or recombination of multiple pre-CTXΦ members with CTXΦ members may result in the emergence of complex CTXΦ family members carrying *ctxAB*.

The members and co-integration arrangements of CTXΦ/pre-CTXΦ prophages in serogroup O139 strains were highly complex compared with those in serogroup O1 strains, presenting the bloom period of the CTXΦ/pre-CTXΦ prophage family during wave 2 of the seventh cholera pandemic. Wave 2 strains can be classified into three clusters (Figure 2). The first cluster is characterized by one or more copies of CTXΦ and the absence of RS1-associated elements. In this cluster, five strains (ICDC-VC0605, 0143, 0129, 4039, and 2370) appeared after 2000, and four O1 strains contain two CTX^class^Φ sequences (*rstR^class^*, *ctxB1*). The three strains (ICD-VC0129, 4039, and 2370) exhibited the presence of the “RS1-RS1” gene structure in Chr. 2. The emergence of this arrangement can be attributed to the infection of class strains by CTX^ET^Φ strains, resulting in the exchange of genes between *rstR^ET^* and *rstR^class^*: first, CTX^ET^Φ infected classical strains, resulting in the loss of CTX^class^Φ; second, CTX^ET^Φ was lost from the large chromosome, allowing the integration of CTX^class^Φ into the small chromosome [37,38]. The second cluster comprises three strains, namely, ICDC-VC1824, 3741, and 1575. Among the pandemic strains, VC1575 has a typical “CTXΦ-RS1” structure of the ET biotype [36] but contains an additional integration of pre-CTX^ZJ^Φ. VC3741 has the skeleton of the seventh pandemic strain, and it is postulated that it may have lost the *ctxAB* gene in a specific environment, thereby becoming a strain that only carries pre-CTXΦ. VC1824 contains RS1 elements exclusively and may have lost pre-CTXΦ based on the genome structure of VC3741. The third cluster contains O139 strains with CTX^ET^Φ, different pre-CTXΦ types, and RS1 elements. ICDC-VC2530 exhibits CTX^ET^Φ-RS1, while the remaining strains have complex CTXΦ arrays. The most prevalent structure is CTX^ET^Φ-RS1-CTX^ET^Φ, followed by the duplication of pre-CTXΦ forms, some of which are present in multiple copies. These results indicate that the serogroup O139 strains exhibited a higher complexity, functional activity, and structural diversity than the serogroup O1 strains, resulting in the dissemination of CTXΦ/pre-CTXΦ family members.

The *rstR* gene binds to the ig-2 region of the *rstAB* promoter and inhibits *rstAB* expression in the same CTXΦ type but cannot inhibit *rstAB* expression in other CTXΦ types [21,22]. The analysis of *rstAB* promoter activity using fluorescent reporter plasmids indicates that the RstR-mediated repression of *rstA* expression is biotype-specific. Upon initial infection with pre-CTXΦ, a strain may become immune to reinfections by the same or other phage types. The presence of multiple *rstR* alleles in individual strains suggests that pre-CTXΦ can coexist with RS1. The presence of several members of the CTXΦ/pre-CTXΦ family may promote the emergence of new virulent strains, underscoring the impact of the integration of pre-CTXΦ elements on the evolution of new strains. We speculate that the co-integration of CTXΦ and pre-CTXΦ occurs through infection events. There was a transient increase in the prevalence of CTXΦ and pre-CTXΦ in wave 2 strains, with diversified CTXΦ replication, genomic integration, and horizontal transfer. This result suggests the occurrence of a cholerae outbreak caused by O139 strains in the Chinese mainland during this wave and the formation of complex co-integration patterns. Pre-CTXΦ may contribute to the diversity of the CTXΦ family by serving as an intermediate pool for *rstR* alleles [31,39].

The present study evaluated the CTXΦ/pre-CTXΦ family, which has high structural diversity and is characterized by different co-integration patterns. These analyses have enhanced our comprehension of the genetic variation of CTXΦ/pre-CTXΦ. Although pre-CTXΦ has been identified as a selfish genetic element, its role in the interaction with *V. cholerae* and its evolutionary impact remain to be understood, particularly given the numerous different alleles within the *V. cholerae* population. Under certain accidental circumstances, this co-integration can present certain risks. The diverse *rstR* alleles facilitate the coexistence of different CTXΦ members within a single strain, providing further evidence for the study of CTXΦ/pre-CTXΦ family evolution.

## 5. Conclusions

This study investigated the gene organization structure, copy number variation, and co-integration pattern of *V. cholerae* CTXΦ/pre-CTXΦ carrying diverse *rstR* alleles, demonstrating the diversity of the CTXΦ/pre-CTXΦ family members, the co-integration of multiple members in chromosomes, and the complex genomic arrangement. A more complex CTXΦ/pre-CTXΦ family form was found in strains from wave 2 of the seventh cholera pandemic compared to strains from waves 1 and 3. Also, the members and co-integration arrangements of CTXΦ/pre-CTXΦ prophages in serogroup O139 strains were highly complex compared with those in serogroup O1 strains. The analysis of *rstAB* promoter activity using reporter plasmids indicates that the RstR allele specifically inhibits the expression of *rstAB*, but cannot inhibit the expression of *rstAB* in other CTXΦ types. These results indicate that when the cholera outbreak was caused by the O139 strains in the Chinese mainland during wave 2, pre-CTXΦ may have promoted the diversity of the CTXΦ family by serving as an intermediate pool of *rstR* alleles. In summary, this study enriches our understanding of the diversity and complex co-integration patterns of CTXΦ/pre-CTXΦ family members. Further, it elucidates the function of *rstR* in the expression and replication of CTXΦ/pre-CTXΦ, establishing a basis for investigating the roles and contributions of CTXΦ/pre-CTXΦ family members in evolutionary processes.

## Figures and Tables

**Table 1:** Isolation and genome structure information of 21 strains of *V. cholerae*.

Strain	Year Isolated	ST	Serogroup	CTXΦ			pre-CTXΦ	
				*rstR* Type	*ctxB* Type	Chr	*rstR* Type	Chr
ICDC-VC2873	1961	69	O1	ET	3	1	-	-
ICDC-VC2874	1961	69	O1	ET	3	1	-	-
ICDC-VC1824	1984	69	O1	-	-	-	-	-
ICDC-VC1575	1990	69	O1	ET	1	1	ET	1
							ZJ, 4**	2
ICDC-VC3741	1990	69	O1	-	-	-	ET	1
ICDC-VC4039	1993	69	O1	class	1	2	-	-
ICDC-VC0129	1994	69	O1	class	1	2	-	-
ICDC-VC2530	1996	69	O1	ET	3	1	ZHJ, 232	2
ICDC-VC0368	2000	69	O139	ET	3	1	ZJ	1
ICDC-VC0605	2000	69	O1	class, ET	1	2	-	-
ICDC-VC0143	2001	69	O1	class	1	2	-	-
ICDC-VC1398	2002	69	O139	ET	3	1	ZJ	1
ICDC-VC0909	2002	69	O139	ET	3	single	ZJ	
ICDC-VC0449	2004	69	O139	ET	3	1	ET	1
							ZHJ	2
ICDC-VC1451	2006	173	O1	-	-	-	ET	1
ICDC-VC1459	2006	173	O1	-	-	-	ZHJ	1
ICDC-VC0636	2006	69	O139	ET	3	1	ZJ	1
ICDC-VC1464	2006	69	O139	ET	3	1	ZJ, 6	1
ICDC-VC2370	2007	69	O1	class	1	2	-	-
ICDC-VC4354	2010	69	O139	ET	3	1	ZJ, 6	1
							ZHJ, 232	2
ICDC-VC5997	2014	69	O139	ET	3	1	232	1

**Table 2:** Luminescence values obtained after coexpression of eight types of pB-*rstR* and pBR-ig2.

Type	*pBR*-ig-2^ET^	*pBR*-ig-2^class^	*pBR*-ig2^232^	*pBR*-ig-2^calc^	*pBR*-ig-2^5^	*pBR*-ig-2^6^	*pBR*-ig-2^ZJ^	*pBR*-ig-2^ZHJ^
vector	5.04 × 10^5^	6.42 × 10^5^	3.32 × 10^5^	6.51 × 10^4^	6.44 × 10^4^	2.18 × 10^5^	6.53 × 10^5^	6.04 × 10^5^
ET	5.57 × 10^4^	2.56 × 10^6^	3.55 × 10^6^	3.28 × 10^5^	3.06 × 10^6^	3.70 × 10^5^	1.05 × 10^6^	2.54 × 10^6^
class	3.56 × 10^6^	1.09 × 10^5^	4.16 × 10^6^	2.58 × 10^6^	4.60 × 10^5^	3.80 × 10^6^	8.03 × 10^6^	3.18 × 10^6^
232	1.72 × 10^6^	2.50 × 10^6^	2.72 × 10^3^	4.95 × 10^4^	1.31 × 10^4^	2.56 × 10^5^	7.12 × 10^5^	2.26 × 10^4^
calc	2.43 × 10^6^	1.04 × 10^6^	3.28 × 10^6^	4.46 × 10^4^	2.19 × 10^6^	2.67 × 10^6^	7.58 × 10^6^	2.25 × 10^6^
5	2.29 × 10^6^	2.60 × 10^6^	1.94 × 10^6^	4.31 × 10^5^	5.53 × 10^4^	2.61 × 10^6^	6.88 × 10^6^	6.03 × 10^5^
6	4.46 × 10^6^	4.46 × 10^6^	1.16 × 10^6^	1.75 × 10^5^	2.29 × 10^5^	1.94 × 10^4^	1.05 × 10^6^	5.17 × 10^6^
ZJ	3.02 × 10^6^	2.95 × 10^6^	1.15 × 10^6^	1.24 × 10^5^	7.02 × 10^6^	2.78 × 10^6^	4.86 × 10^4^	3.85 × 10^6^
ZHJ	3.07 × 10^6^	2.04 × 10^6^	4.75 × 10^6^	3.23 × 10^5^	3.11 × 10^6^	1.08 × 10^6^	1.04 × 10^7^	1.20 × 10^5^

## Data Availability

Data are deposited in the National Microbiology Data Center (NMDC) with accession number NMDC10019036 (https://nmdc.cn/resource/genomics/project/detail/NMDC10019036, accessed on 25 July 2024).

## Supplementary Materials

The following supporting information can be downloaded at: https://www.mdpi.com/article/10.3390/microorganisms12101935/s1, Table S1: Strains and plasmids used in this study; Table S2: Primers used in this study; Table S3: GenBank accession numbers; Table S4. Difference in the sequences of *rstA* and *rstB* genes within CTXΦ were identified in 7PC *V. cholerae;* Table S5: Metadata of *V. cholerae.*
