# Supplementary material for: Diversity and Complexity of CTXΦ and Pre-CTXΦ Families in Vibrio cholerae from Seventh Pandemic
Source: Microorganisms. 2024 Sep 24;12(10):1935. doi: 10.3390/microorganisms12101935 (PMC11509585; doi:10.3390/microorganisms12101935)
Supplement: Supplementary file 1 [file microorganisms-12-01935-s001.zip › SupplementrayS1-S4.pdf]

Diversity and Complexity of CTXΦ and Pre-CTXΦ Families in *Vibrio cholerae* from Seventh Pandemic

Table S1. Strains and plasmids used in this study

Strain and Plasmid	Characteristics	References and Sources
E. coli		
SM10*λpir*	*supE recA*: :RP4-2Tc: :Mu;Km^r	Lab stock
DH5α*λpir*	F-D(*lacZYA-argF*)U169 *recA endA1 supE44 relA1λ::pir*	Lab stock
JM109	*recA1, endA1, hsdR17, supE44, gyrA96,thiΔ(lac-proAB)F'[traD36, proAB+, $lacI^q$, lacZΔM15]*	TaKaRa
V. cholerae		
N16961	O1, $CTX^{ET}Φ$, *$rstR^{ET}$*, *$ctxAB^+$*, *$tcpA^+$*	Lab stock
1119	O1, Inaba,*$rstR^{Class}$*, $CTX^{Class}Φ^+$, tcpA+	Lab stock
ICDC-VC2873	O1, Ogawa,$CTX^{ET}Φ$,*$rstR^{ET}$, $ctxAB^+$, $tcpA^+$*	Lab stock
ICDC-VC2874	O1, Ogawa,$CTX^{ET}Φ$, *$rstR^{ET}$,$ctxAB^+$, $tcpA^+$*	Lab stock
ICDC-VC1824	O1, Ogawa,RS1*, $tcpA^+$*	Lab stock
ICDC-VC1575	O1, Ogawa,*$rstR^{ET}$,$rstR^{ZJ}$*,RS1, $CTX^{ET}Φ$, pre-$CTX^{ZJ}Φ$, *$ctxAB^+$, $tcpA^+$*	Lab stock
ICDC-VC3741	O1, Ogawa,pre-$CTX^{ET}Φ$,RS1,*$rstR^{ET}$ $tcpA^+$*	Lab stock
ICDC-VC4039	O1, Ogawa,RS1,$CTX^{Class}Φ$,*$rstR^{Class}$, $ctxAB^+$, $tcpA^+$*	Lab stock
ICDC-VC0129	O1, Ogawa, RS1,$CTXC^{Class}Φ$,*$rstR^{Class}$, $ctxAB^+$, $tcpA^+$*	Lab stock
ICDC-VC2530	O1, Ogawa,*$rstR^{ET/ZJ}$*,RS1, $CTX^{ET}Φ$, pre-$CTX^{ZJ}Φ$, *$ctxAB^+$, $tcpA^+$*	Lab stock
ICDC-VC0386	O139, *$rstR^{ET/ZJ}$*,RS1, $CTX^{ET}Φ$, *$ctxAB^+$, $tcpA^+$*	Lab stock
ICDC-VC0605	O1, Ogawa,*$rstR^{ET/ZJ}$*RS1, $CTX^{ET}Φ$, pre-$CTX^{ZJ}Φ$, *$ctxAB^+$, $tcpA^+$*	Lab stock
ICDC-VC0143	O1, Ogawa, RS1,$CTX^{Class}Φ$,*$rstR^{Class}$, $ctxAB^+$, $tcpA^+$*	Lab stock
ICDC-VC0909	O139, *$rstR^{ET}$*,RS1, $CTX^{ET}Φ$,*$ctxAB^+$, $tcpA^+$*	Lab stock
ICDC-VC1398	O139, *$rstR^{ET/ZJ/6}$*, RS1, $CTX^{ET}Φ$,pre-$CTX^{ZJ/6}Φ$,*$ctxAB^+$, $tcpA^+$*	Lab stock
ICDC-VC0449	O139, *$rstR^{ET/ZHJ}$*, RS1, $CTX^{ET}Φ$,pre-$CTX^{ZHJ/ET}Φ$,*$ctxAB^+$, $tcpA^+$*	Lab stock
ICDC-VC0636	O139, *$rstR^{ZJ/ET/4/ZHJ}$*, RS1, $CTX^{ET}Φ$,pre-$CTX^{ZJ/4/ZHJ}Φ$,*$ctxAB^+$, $tcpA^+$*	Lab stock
ICDC-VC1451	O1,Inaba, *$rstR^{calc}$*, RS1,pre-$CTX^{calc}Φ$, *$tcpA^+$*	water

ICDC-VC1459	O1,Inaba, *rstR*^{ZHJ}, RS1,pre-CTX^{ZHJ}Φ, *tcpA*$^{+}$	water
ICDC-VC1464	O139, *rstR*$^{ZJ/ET/6}$, RS1, CTX^{ET}Φ,pre-CTX$^{ZJ/6}$Φ,*ctxAB*$^{+}$, *tcpA*$^{+}$	Lab stock
ICDC-VC2370	O139, *rstR*$^{ET/Class}$, RS1, CTX^{Class}Φ,*ctxAB*$^{+}$, *tcpA*$^{+}$	Lab stock
ICDC-VC4354	O139, *rstR*$^{ET/ZJ/6}$, RS1, CTX^{ET}Φ,pre-CTX$^{ZJ/6}$Φ,*ctxAB*$^{+}$, *tcpA*$^{+}$	Lab stock
ICDC-VC5997	O139, *rstR*$^{ET/232}$, RS1, CTX^{ET}Φ,pre-CTX^{232}Φ,*ctxAB*$^{+}$, *tcpA*$^{+}$	Lab stock
Plasmids		
pBAD24(pB)	pBR322 *ori*, *araC*, *bla*, Amp^{r}	Lab stock
pBBR*lux*(pBR)	promoterless of *lux*CDABE, Cm^{r}	Lab stock
pB-*rstR*-ET	pBAD24 containing 339bp *rstR*^{ET}, Amp^{r}	This study
pB-*rstR*-Class	pBAD24 containing 336bp *rstR*^{Class}, Amp^{r}	This study
pB-*rstR*-Calc	pBAD24 containing 180bp *rstR*^{Calc}, Amp^{r}	[1]
pB-*rstR*-232	pBAD24 containing 345bp *rstR*^{232}, Amp^{r}	[2]
pB-*rstR*-5	pBAD24 containing 204bp *rstR*^{5}, Amp^{r}	[3]
pB-*rstR*-6	pBAD24 containing 192bp *rstR*^{6}, Amp^{r}	[4]
pB-*rstR*-*ZJ*	pBAD24 containing 354bp *rstR*^{ZJ}, Amp^{r}	[5]
pB-*rstR*-ZHJ	pBAD24 containing 333bp *rstR*^{ZHJ}, Amp^{r}	[6]
pBR-*ig2*-ET	pBBR*lux* containing *ig2*^{ET}, Cm^{r}	This study
pBR-*ig2*-Class	pBBR*lux* containing *ig2*^{Class}, Cm^{r}	This study
pBR-*ig2*-Calc	pBBR*lux* containing *ig2*^{Calc}, Cm^{r}	[1]
pBR-*ig2*-232	pBBR*lux* containing *ig2*^{232}, Cm^{r}	[2]
pBR-*ig2*-5	pBBR*lux* containing *ig2*^{5}, Cm^{r}	[3]
pBR-*ig2*-6	pBBR*lux* containing *ig2*^{6}, Cm^{r}	[4]
pBR-*ig2*-ZJ	pBBR*lux* containing *ig2*^{ZJ}, Cm^{r}	[5]
pBR-*ig2*-ZHJ	pBBR*lux* containing *ig2*^{ZHJ}, Cm^{r}	[6]

Table S2. **Primers used in this study**

Primer pairs	Oligonucleotide sequences (5'-3') *	purposes
$rstR^{ET}$-F	ATGAAGATAAAAGAAAGGCTAGCCAACC	*$rstR^{ET}$*
$rstR^{ET}$-R	CTAAGCACCATGATTTAAGATGCTCTTG	
$rstR^{class}$-F	ATGTTTAGTTCAAAAATTAGGGATTTAAG	*$rstR^{class}$*
$rstR^{class}$-R	CTACCTAAATTCTTTTTGTATTTCTCGAC	
$rstR^{calc}$-F	ATGGCAACAAAGCACATTAAAGACAGTACG	*$rstR^{calc}$*
$rstR^{calc}$-R	GTCAAGCTTTTTTTTGCTTTATCTTATGGAAG	
$rstR^{232}$-F	ATGCTTAGAGATAAGATTAAACAGTGTCG	*$rstR^{232}$*
$rstR^{232}$-R	TTAGCTTAGGTTTTTGAATTTTGCTTTAGC	
$rstR^{5}$-F	ATGGATGTATCTAAAACCAGTCTCG	*$rstR^{5}$*
$rstR^{5}$-R	TTACTTTGTTAGTTCTTCTGTTTTTCCG	
$rstR^{6}$-F	ATGAAAAAAAAAATACACATCACTAAGAGTTAG	*$rstR^{6}$*
$rstR^{6}$-R	TTAACGCTTTAAGTCCGTTTTTTTGTGTATTAG	
$rstR^{ZJ}$-F	ATGGGGGATGAAATGATTAATGCG	*$rstR^{ZJ}$*
$rstR^{ZJ}$-R	TTAATTTATGCTTAATTCATTTCTAGCTTTATTGC	
$rstR^{ZHJ}$-F	ATGATTGGTGAAAAAATAAAAGCACTTAGAGAG	*$rstR^{ZHJ}$*
$rstR^{ZHJ}$-R	CTATAAGGTTTTAGTTAGCTCGATGTTCTGTC	
$rstR^{ET}$-F-EcoRI	TTTGGGCTAGCAGGAGGAATTCATGAAGATAAAAGAAAGGC	construct the recombinant plasmid
$rstR^{ET}$-R-XbaI	GCCTGCAGGTCGACTCTAGACTAAGCACCATGATTTAAG	pB-*rstR*-ET
$rstR^{class}$-F-EcoRI	TTTGGGCTAGCAGGAGGAATTCATGTTTAGTTC	construct the recombinant plasmid
$rstR^{class}$-R-XbaI	GCCTGCAGGTCGACTCTAGACTACCTAAATTCTTTTTGTATTTCTC	pB-*rstR*-Class
$rstR^{calc}$-F-NcoRI	GGAGGAATTCACCATGGATGGCAACAAAGCACATTAAAGAC	construct the recombinant plasmid
$rstR^{calc}$-R-SalI	GCATGCCTGCAGGTCGACTCAAGCTTTTTTTTGCTTTATCTTATG	pB-*rstR*-Calc
$rstR^{232}$-F-EcoRI	GGGCTAGCAGGAGGAATTCATGCTTAGAGATAAG	construct the recombinant plasmid
$rstR^{232}$-R-XbaI	CTGCAGGTCGACTCTAGATTAGCTTAGGTTTTTGAATTTTG	pB-*rstR*-232
$rstR^{5}$-F-EcoRI	GGGCTAGCAGGAGGAATTCATGGATGTATCTAAAACC	construct the recombinant plasmid
$rstR^{5}$-R-XbaI	GCCTGCAGGTCGACTCTAGATTACTTTGTTAGTTCTTC	pB-*rstR*-5
$rstR^{6}$-F-EcoRI	CTAGCAGGAGGAATTCATGAAAAAAAAAATACACATCACTAAG	construct the recombinant plasmid
$rstR^{6}$-R-XbaI	CTGCAGGTCGACTCTAGATTAACGCTTTAAGTCCGTTTTTTTG	pB-*rstR*-6
$rstR^{ZJ}$-F-EcoRI	GGGCTAGCAGGAGGAATTCATGGGGGATGAAATG	construct the recombinant plasmid
$rstR^{ZJ}$-R-XbaI	GCCTGCAGGTCGACTCTAGATTAATTTATGCTTAATTCATTTC	pB-*rstR*-ZJ
$rstR^{ZHJ}$-F-NcoRI	GGAATTCACCATGGATGATTGGTGAAAAAATAAAAGC	construct the recombinant plasmid
$rstR^{ZHJ}$-R-SalI	GCATGCCTGCAGGTCGACCTATAAGGTTTTAGTTAGCTC	pB-*rstR*-ZHJ
$ig2^{ET}$-F-SacII	TATAGGGCGAATTGGAGCTCTAGCCTTCAAAAACCTGTC	construct the recombinant plasmid
$ig2^{ET}$-R-BamHI	GCAACTAGAGGATCCAACAATCCCCTTTAGCTTG	pBR-*ig2*-ET
$ig2^{class}$-F-SacII	GAATTGGAGCTCACATACCACCCCTATAG	construct the recombinant plasmid
$ig2^{class}$-R-BamHI	CGCAACTAGAGGATCCTAGCCTTC	pBR-*ig2*-Class
$ig2^{calc}$-F-SacII	GCGAATTGGAGCTCTGTTTAACCTCTCTTG	construct the recombinant plasmid
$ig2^{calc}$-R-BamHI	CAACTAGAGGATCCACCAAAACCCACAAAAC	pBR-*ig2*-Calc
$ig2^{232}$-F-SacII	GGCGAATTGGAGCTCTTAACTTTCCCCCTATG	construct the recombinant plasmid
$ig2^{232}$-R-BamHI	CGCAACTAGAGGATCCGCACCAAAAACCTGTC	pBR-*ig2*-232
$ig2^{6}$-F-SacII	GGCGAATTGGAGCTCTTTTAACTTCCTATTGC	construct the recombinant plasmid

ig2^{6}-R-BamHI	GCAACTAGAGGATCCAGATAACTCATTAATGC	pBR-*ig2*-6
ig2^{ZJ}-F-SacII	GGCGAATTGGAGCTCGTTTGGTTTAATTATTTTCCC	construct the recombinant plasmid
ig2^{ZJ}-R-BamHI	GCAACTAGAGGATCCGAGGAAATATTTTTCATAATC	pBR-*ig2*-ZJ
ig2^{ZHJ}-F-SacII	CTAGAGCTCCTCGTATTTTATGTATGTTG	construct the recombinant plasmid
ig2^{ZHJ}-R-BamHI	ATCGGATCCGCACCAAAAACCTGTCAAG	pBR-*ig2*-ZHJ

*The underlined bases indicate the restriction enzyme sites.

Table S3. GenBank accession numbers

**GenBank**	Strain
KP768424	V06-18(ZJ)
AF133307	SCE223(4**)
AF133309	SCE263(4)
AF110029	AS207(calc)
AF452586	506-94(6)
DQ288668	VCE232(232)
AF319656	SCE263(5)
AE003852	N16961(ET)
AF055890	569B(Class)

Table S4. Difference in the sequences of *rstA* and *rstB* genes within CTX phage were identified in 7PC *V. cholerae*.

CTX type	*rstR* type	*ctxB*	*rstA* sequence							*rstB* sequence						
			27	162	183	258	927	933	942	74-76	87	93	105	189	360	364
CTX-1	El Tor	3	C	C	C	G	T	C	G	GTA	A	T	G	A	A	C
CTX-2	Class	1	T	T	A	C	T	C	G	△	T	C	G	A	A	C
CTX-3	El Tor	1	C	C	C	G	C	T	T	GTA	A	T	G	A	A	C
CTX-3b	El Tor	7	C	C	C	G	C	T	T	GTA	A	T	G	A	A	C
CTX-4	El Tor	1	T	T	A	C	T	C	G	△	A	T	G	A	A	C
CTX-5	El Tor	1	T	T	A	C	T	C	G	△	T	C	A	A	A	C
CTX-6	El Tor	1	T	T	A	C	T	C	G	△	T	C	A	G	A	C
CTX-6b	El Tor	1	T	T	A	C	T	C	G	△	T	C	A	G	A	C
CTX-O139	Calc	3	C	C	C	G	T	C	G	GTA	A	T	G	A	A	C

[1] DAVIS BRIGID M, KIMSEY HARVEY H, CHANG W, et al. The Vibrio cholerae O139 Calcutta Bacteriophage CTXφ Is Infectious and Encodes a Novel Repressor [J]. Journal of Bacteriology, 1999, 181(21): 6779-87.

[2] MAITI D, DAS B, SAHA A, et al. Genetic organization of pre-CTX and CTX prophages in the genome of an environmental Vibrio cholerae non-O1, non-O139 strain [J]. Microbiology, 2006, 152(12): 3633-41.

[3] MUKHOPADHYAY A K, CHAKRABORTY S, TAKEDA Y, et al. Characterization of VPI pathogenicity island and CTXφ prophage in environmental strains of Vibrio cholerae [J]. Journal of bacteriology, 2001, 183(16): 4737-46.

[4] LI M, KOTETISHVILI M, CHEN Y, et al. Comparative genomic analyses of the vibrio pathogenicity island and cholera toxin prophage regions in nonepidemic serogroup strains of Vibrio cholerae [J]. Applied and Environmental Microbiology, 2003, 69(3): 1728-38.

[5] WANG H, PANG B, XIONG L, et al. The Hybrid Pre-CTXΦ-RS1 Prophage Genome and Its Regulatory Function in Environmental Vibrio cholerae O1 Strains [J]. Appl Environ Microbiol, 2015, 81(20): 7171-7.

[6] LI X, ZHAO L, GAO H, et al. A novel pre-CTX prophage in the Vibrio cholerae serogroup O139 strain [J]. Infection, Genetics and Evolution, 2020, 81(104238.
